# Congenital *in situ* malrotation of the liver in an asymptomatic adult: a rare entity

**DOI:** 10.1259/bjrcr.20150426

**Published:** 2016-10-14

**Authors:** Koushik Sarkar, Jayati Bardhan, Sujata Sarangi

**Affiliations:** ^1^Department of Radiodiagnosis, Bankura Sammilani Medical College, Bankura, India; ^2^Department of Pathology, R G Kar Medical College, Kolkata, India

## Abstract

Congenital malformations of the liver are rare occurrences. We are reporting a case of *in* *situ* malrotation of the liver. The patient was asymptomatic and had undergone a non-contrast CT scan of the upper abdomen, which showed malrotation of the liver. The purpose of submitting this case report is to make radiologists and surgeons aware of this unusual anatomical variation. Malrotation of the liver as a part of heterotaxy syndrome or situs ambiguous has been reported, but isolated malrotation of the liver without polysplenia in an adult male is a rare entity. A similar case has been reported in the literature as an incidental autopsy finding. Relevant references to this case are given below.

## Summary

Congenital anomalies of the liver may pose problems for radiologists and surgeons. Lobar anomalies such as an accessory lobe or situs abnormalities are usually found as an incidental finding in all age groups during surgeries^1^ or autopsies.^2^ Herein we report a case of isolated malrotation of the liver in an adult male, which is relevant for gallbladder-related surgery.

## Case report

**Figure 1.**
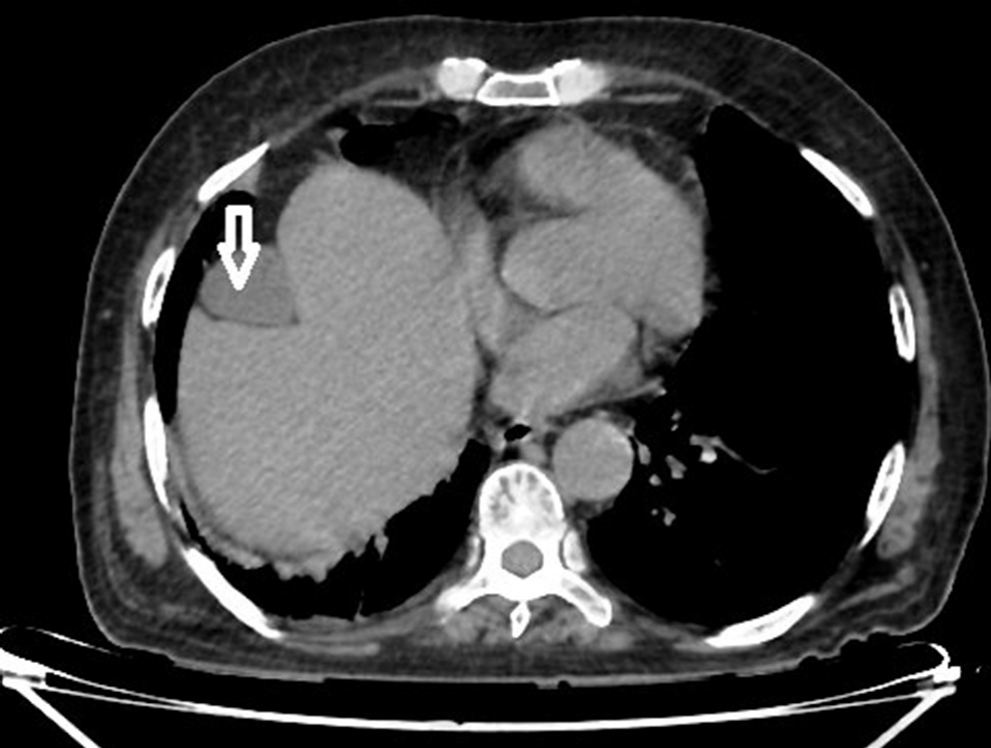
Axial non-contrast CT scan showing the gallbladder (white arrow), which is facing anteriorly.

**Figure 2.**
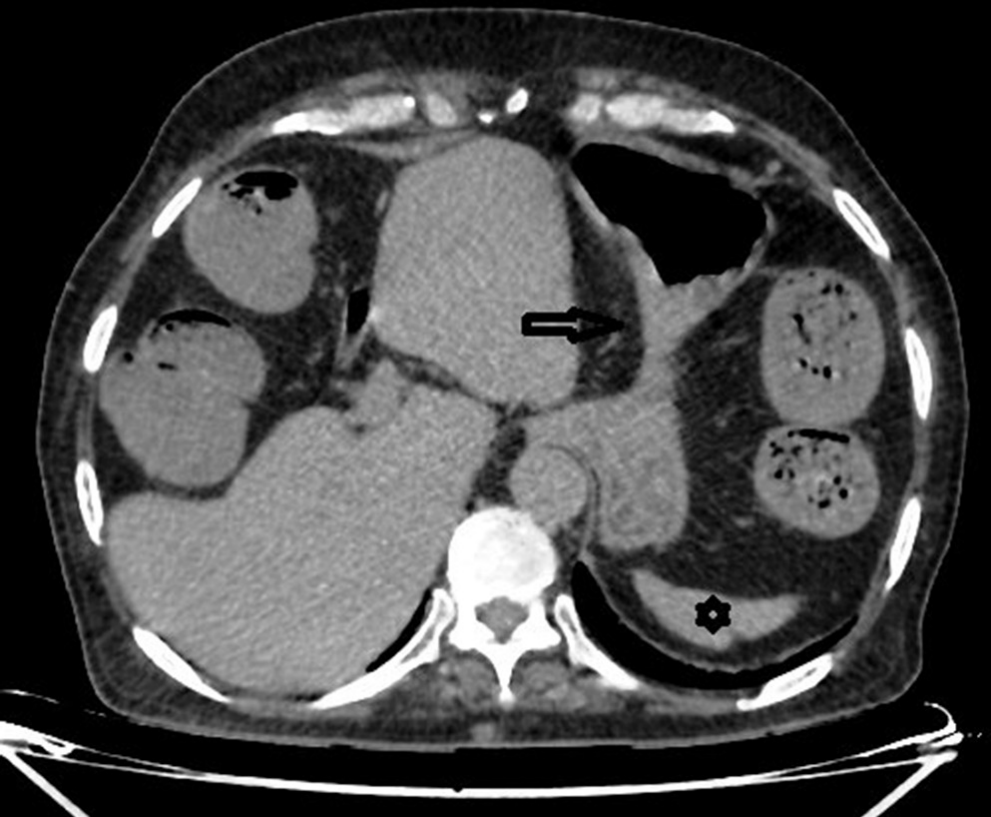
Axial non-contrast CT scan showing the stomach (black arrow) and the spleen (black star) on the left side.

**Figure 3.**
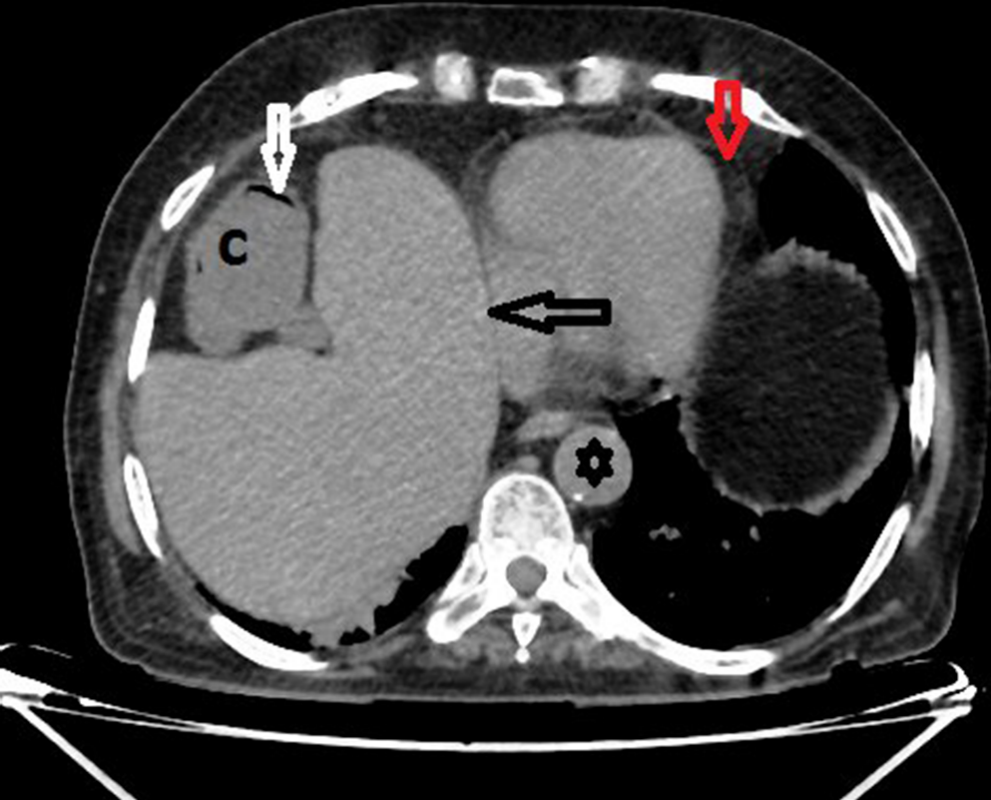
Axial non-contrast CT scan showing the cardiac apex (vertical red arrow) directed towards the left and the large intestine (C) with colonic air (white arrow) in a non-dependent position. The aorta (black star) and the posterior border of the liver (horizontal black arrow) are also seen.

**Figure 4.**
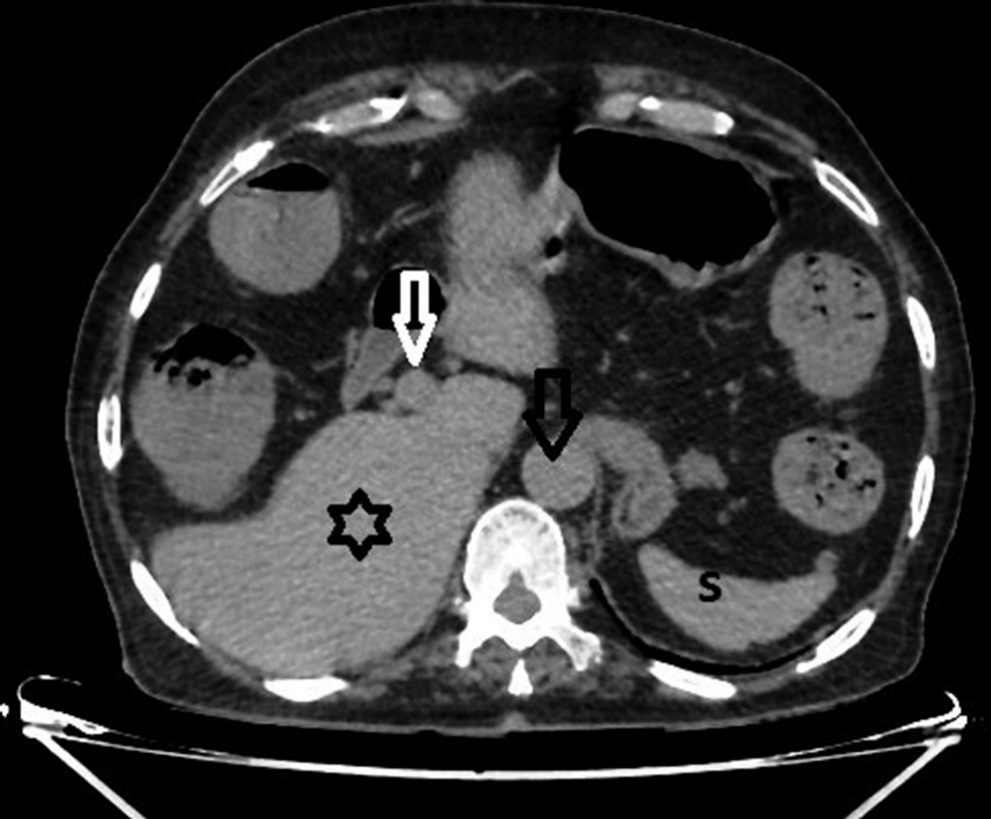
Axial non-contrast CT scan showing the abdominal aorta (black arrow), spleen (S), portal vein (white arrow) and liver (black star). A noteworthy feature is the absence of the inferior vena cava from its normal anatomical position.

**Figure 5.**
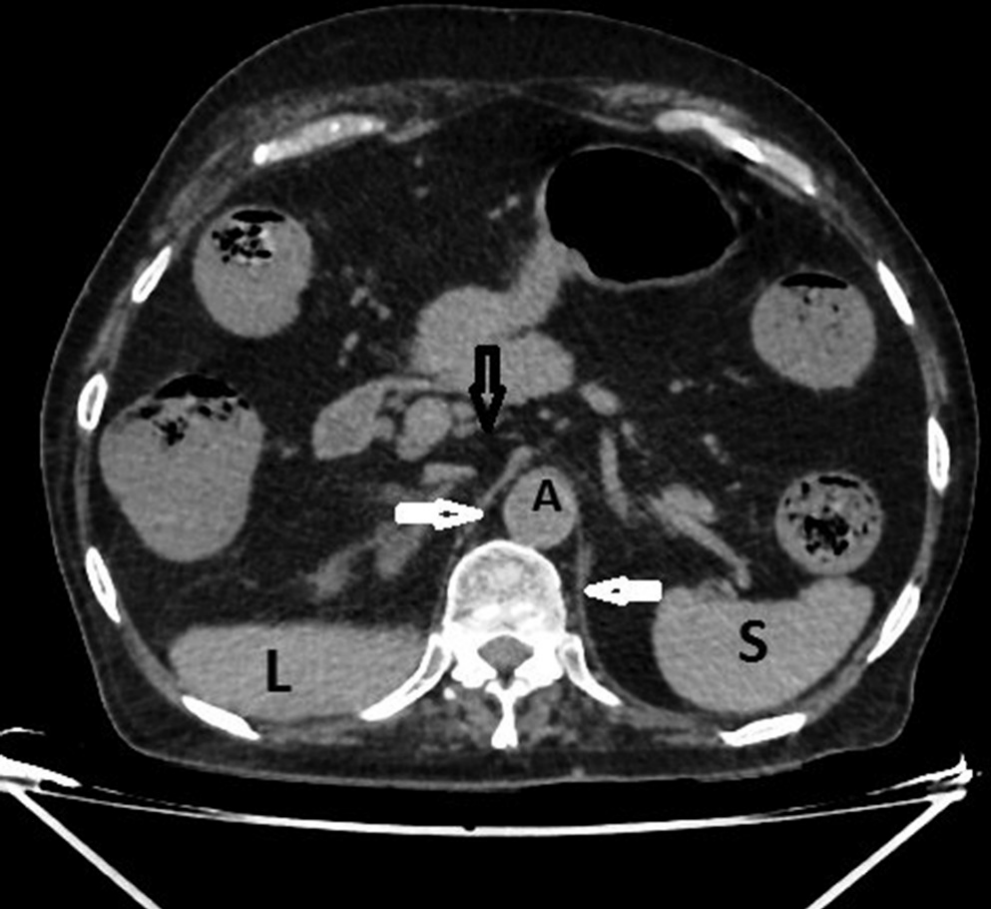
Axial non-contrast CT scan showing the abdominal aorta (A) and the normal position of the inferior vena cava (black arrow), which is empty owing to the absence of the inferior vena cava from its normal anatomical position. The crura of the diaphragm (white arrows), and the liver (L) and spleen (S) are also seen.

An 82-year-old Indian male presented to the surgical outpatient department with complaints of non-specific left flank pain. During the course of the investigations, a non-contrast CT scan was performed, which was unremarkable except for the liver. The liver appeared to be malrotated *in* *situ*, with the inferior surface and the gallbladder facing anteriorly (Figure 1). The left lobe appeared to be atrophic and the right lobe showed physiological hypertrophy. The situs of the patient was normal. The left atrium, spleen and stomach were located on the left (Figure 2) and the right atrium and liver on the right side, with the cardiac apex pointing towards the left side (Figure 3). No evidence of polysplenia or asplenia was seen. There was a single spleen, which was normal in size and position (Figure 4). Another interesting finding was the abnormal position of the inferior vena cava, which was not seen on the right side of the abdominal aorta (Figure 5). No contrast was given as the patient was old. He was subsequently lost to follow-up.

## Discussion

From the middle of the third to early fourth week of development of a fetus, the hepatic diverticulum appears at the distal end of the foregut as an endodermal epithelium. The liver bud, after penetrating the septum transversum, divides into the right and left parts, which ultimately give rise to individual lobes. The pars cystica, which gives rise to the gallbladder and cystic duct, is a ventral outgrowth from the developing bile ducts. The bile duct, which initially opens in the developing duodenum ventrally owing to rotation of the gut, eventually opens dorsally. Anatomical variation of the liver is a rare incidental finding, with very few of them posing any physiological or functional limitations to the organ. Lobar or segmental agenesis or fusion constitutes the majority of anatomical variations.^1^ Usually anatomical variations related to situs anomalies are more commonly found in the paediatric population.^3^ Liver malrotation is an extremely rare event. Only two cases have been reported in the literature. One was in a patient with antiphospholipid antibody syndrome as an incidental post-mortem finding by Zhong,^4^ and the other in an adult with congenital diaphragmatic hernia.^5^ To the best of our knowledge, no such finding has been reported in a living patient. Owing to the patient's old age and financial constraints, a contrast study was not performed and we lost the patient to follow-up.

## Conclusions

Anatomical variations of the liver as well as other gut structures should be noted and kept in mind before any operative procedure of the abdomen and pre-operative evaluation of such conditions is extremely useful. With recent advances in radiology and the increasing use of investigative procedures, radiologists should be aware of such anatomical variations. The occurrence of such a rare malrotation of the liver in a living subject makes this report a unique one.

## Learning points

1. Anatomical variation of the liver is a rare entity and the majority of cases are seen with some rotational abnormalities of the gut.
2. Isolated malrotation is an extremely rare condition wherein the inferior surface of the liver and the gallbladder face anteriorly.
3. These conditions can create problems for clinicians and radiologists in making a diagnosis.
4. Cholecystectomy, whether open or laparoscopic, needs a special incision as well as approach and the surgeon should be aware of the exact anatomy of the biliary tract in the patient. Hence preoperative MR cholecystopancreatography is necessary.
5. Clinicians and radiologists should always look for signs and symptoms of associated congenital anomalies such as gut malrotation, congenital diaphragmatic hernia, etc.
